# Light-Addressable Measurement of *in Vivo* Tissue Oxygenation in an Unanesthetized Zebrafish Embryo via Phase-Based Phosphorescence Lifetime Detection

**DOI:** 10.3390/s150408146

**Published:** 2015-04-08

**Authors:** Shih-Hao Huang, Chu-Hung Yu, Yi-Lung Chien

**Affiliations:** 1 Department of Mechanical and Mechatronic Engineering, National Taiwan Ocean University, Keelung 202-24, Taiwan; E-Mails: jack00985@hotmail.com (C.-H.Y.); jl0087@yahoo.com.tw (Y.-L.C.); 2 Center for Marine Mechatronic Systems, CMMS, National Taiwan Ocean University, Keelung 202-24, Taiwan

**Keywords:** zebrafish, oxygen, digital micromirror device, phosphorescence lifetime

## Abstract

We have developed a digital light modulation system that utilizes a modified commercial projector equipped with a laser diode as a light source for quantitative measurements of *in vivo* tissue oxygenation in an unanesthetized zebrafish embryo via phase-based phosphorescence lifetime detection. The oxygen-sensitive phosphorescent probe (Oxyphor G4) was first inoculated into the bloodstream of 48 h post-fertilization (48 hpf) zebrafish embryos via the circulation valley to rapidly disperse probes throughout the embryo. The unanesthetized zebrafish embryo was introduced into the microfluidic device and immobilized on its lateral side by using a pneumatically actuated membrane. By controlling the illumination pattern on the digital micromirror device in the projector, the modulated excitation light can be spatially projected to illuminate arbitrarily-shaped regions of tissue of interest for *in vivo* oxygen measurements. We have successfully measured *in vivo* oxygen changes in the cardiac region and cardinal vein of a 48 hpf zebrafish embryo that experience hypoxia and subsequent normoxic conditions. Our proposed platform provides the potential for the real-time investigation of oxygen distribution in tissue microvasculature that relates to physiological stimulation and diseases in a developing organism.

## 1. Introduction

The ability to quantitatively detect oxygenation levels in tissue is important because it can provide physiological information about impaired tissues that can be further referred to planning of clinical therapeutics and assessment of curative efficacy. For examples, a low tissue oxygen concentration, followed by the reintroduction of oxygen, gives rise to a phenomenon known as ischemia/reperfusion (IR) injury, which is harmful for tissues, causing necrotic or apoptotic cell death [1]. Recently, zebrafish (*Danio rerio*) embryos have been proven a suitable model organism to serve as a dynamic model of anoxia tolerance during development [2,3,4], and to study the tissue resistance to hypoxia/reperfusion (HR) [5,6] due to its small size, optical transparency of complex organs, and ease of culture for high-throughput analysis. The methodologies of these investigations mostly focus on the survival, heart beat rates, or genetic/proteomic analysis of zebrafish embryos during HR. The oxygen variations in tissues at the microvascular level in zebrafish embryos during HR have not received much attention in the past literature due to the lack of suitable technologies to monitor the phenomenon. Therefore, the development of a technology to quantitatively detect *in vivo* tissue oxygenation of a zebrafish embryo is highly desirable for investigation of oxygen delivery in tissues in real time at the microvascular level. 

Oxygen-dependent quenching of phosphorescence has recently emerged as a useful and essentially noninvasive optical method for *in vivo* oxygen measurement in model animals [7,8,9,10,11,12,13,14,15,16,17,18,19]. Quantification of oxygen concentrations with oxygen-sensitive phosphorescent dyes is measured by either the degree of luminescent intensity quenching or the luminescent lifetime. Detecting the luminescent lifetime to quantify oxygen concentrations has been proven to have a higher sensitivity due to the inherent stability of the signal. The lifetime-based phosphorescence measurement has been used to effectively measure pO_2_ in many different types of tissues including the brain, retina, tumor and muscle in rats/mice [7,8,11,12,13], microcrustacean *Daphnia magna* [9], chicken embryos [16], and pigs [10,15,17,20]. To acquire the pO_2_ images of the tissue or tumors in model animals, scanning lasers excitation sources [21] or extremely sensitive cameras with gated exposure times [8,9] are typically utilized to achieve spatial resolution. A major drawback of these techniques is the increased time needed to either scan for multiple point measurements or perform significant averaging to improve the poor signal to noise ratios (S/N) of cameras. Dunn *et al*. [11] proposed a method retain the high temporal resolution and sensitivity of single point detection of phosphorescence by using a digital micromirror device (DMD) to selectively illuminate arbitrarily shaped regions of tissue. They demonstrate the usefulness of this system for dynamic and simultaneous measurement of blood flow and pO_2_ in the rat brain via time-domain phosphorescence lifetime measurements. However, the time-domain lifetime measurement inevitably requires high-speed (~10 MHz) data acquisition hardware to directly measure the luminescent decay time. Although the technologies to quantitatively detect *in vivo* tissue oxygenation in animals (rats, pigs, and chicken embryos) have been proposed in the abovementioned studies, none of reported current research referred to a physiologically relevant whole organism model, the zebrafish embryo, probably due to the lack of suitable methods to immobilize the small zebrafish embryos and concurrently perform *in vivo* oxygen measurements under physiological treatment conditions. 

In this study, we proposed a digital light modulation system that utilizes a modified commercial DMD projector equipped with a fiber-guided laser diode as a light modulation source to modulate the excitation light in the spatial and temporal domains for quantitative measurements of *in vivo* tissue oxygenation in an unanesthetized zebrafish embryo in real time via phase-based phosphorescence lifetime detection. The unanesthetized zebrafish embryo was introduced into the microfluidic device and immobilized on its lateral side by using a pneumatically actuated membrane. In contrast to current immobilization methods to embed zebrafish embryos within agarose in a glass capillary [22] where no flow can be introduced passing through embryos, our approach enables us to controllably immobilize embryos and perfuse a normoxic/hypoxic embryo medium into a microfluidic device for long-term oxygen measurements to study the tissue resistance under HR. Besides, the anesthetization of the measured zebrafish embryo is no longer needed to prevent interference with its physiological state. The oxygen-sensitive phosphorescent probe (Oxyphor G4) was inoculated into the bloodstream of 48 h post-fertilization (48 hpf) zebrafish embryos via the circulation valley to rapidly disperse probes throughout the embryo. Phase-based (frequency-domain) phosphorescence lifetime detection was selected as the preferred detection scheme for *in vivo* oxygen measurements. The phosphorescence lifetime was calculated by measuring the phase shift between the reference (modulated excitation light) and corresponding phosphorescent signals to directly measure the luminescent decay time without the need for high-speed data acquisition hardware or complicated and expensive facilities. By controlling the illumination pattern on the DMD, the modulated excitation light can be spatially projected to illuminate arbitrarily-shaped regions of tissue of interest for *in vivo* oxygen measurements. This approach retains the high S/N ratio of single point pO_2_ measurements, but also enables spatial mapping with enough spatial resolution to clearly distinguish different regions.

## 2. Experimental Section 

### 2.1. Principle of Operation 

**Figure 1:**
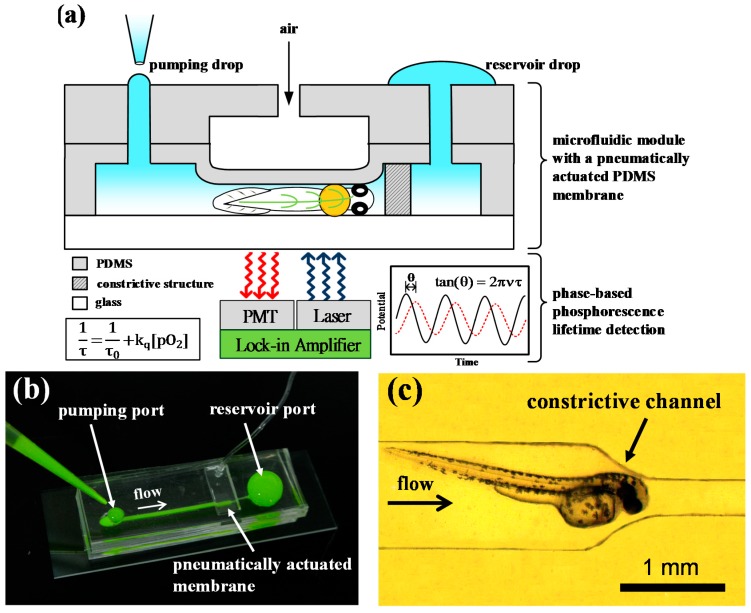
(**a**) A schematic of a microfluidic device to detect *in vivo* tissue oxygenation of an unanesthetized zebrafish embryo in real time via phase-based phosphorescence lifetime detection. The unanesthetized zebrafish embryo was introduced into the microfluidic device via the passive pumping and immobilized on its lateral side by using a pneumatically actuated membrane; (**b**) The image of the microfluidic device with a constrictive channel and a pneumatically actuated membrane, where the fluid can be pumped from the pumping port to the reservoir port by continuously adding small pumping drops at the pumping port; (**c**) The image of an unanesthetized zebrafish embryo which was pumped into the microchannel head-first and then trapped by constrictive channels.

The microfluidic device consisted of a microfluidic channel with constriction to trap an unanesthetized zebrafish embryo and a pneumatically actuated membrane to immobilize the zebrafish embryo on its lateral side for *in vivo* oxygen measurements as illustrated in Figure 1. To perform *in vivo* oxygen measurements, we first inoculated the oxygen-sensitive phosphorescent probe (Oxyphor G4) into the bloodstream of 48 hpf zebrafish embryos via the circulation valley located near to the yolk sac to rapidly disperse probes throughout the embryo via blood circulation. The Oxyphor G4 can operate in albumin-rich (blood plasma) environments and intend to stay in blood circulation without extravasating through the vascular membrane. The hatched zebrafish embryo (48 hpf) was chosen due to its fully development of eyes, brain, notochord, and the cardiovascular system as well as high viability after microinjection with Oxyphor G4. Briefly, the microfluidic device was initially filled by adding embryo water to one port and aspirating from another port until water was drawn into the entire channel. We took an unanesthetized zebrafish embryo into the pipette tail-first and added it into the pumping port of the microfluidic device (Figure 1). Passive pumping [23,24] was used to load an embryo into the channel via surface tension difference. The fluid can be pumped through a microchannel from the pumping port to the reservoir port by continuously adding small pumping drops at the pumping port (Figure 1b). The unanesthetized zebrafish embryo was pumped into the microchannel head-first and then trapped by constrictive microfluidic channels (Figure 1c). After trapping the zebrafish embryo, an air-pressure system, described in our previous work [25,26], was used to pneumatically actuate the PDMS membrane to immobilize the unanesthetized embryo on its lateral side. A regulated compressed-air source was connected to multiple three-way solenoid valves (Lee Inc., Westbrook, CT, USA) to switch rapidly between atmospheric and input pressure, where high pressure air was used to press the PDMS membrane. The immobilization of the unanesthetized zebrafish embryo by using a pneumatically actuated membrane is necessary in our experiments to prevent from free movement of the unanesthetized zebrafish embryo during *in vivo* oxygen measurements. 

**Figure 2:**
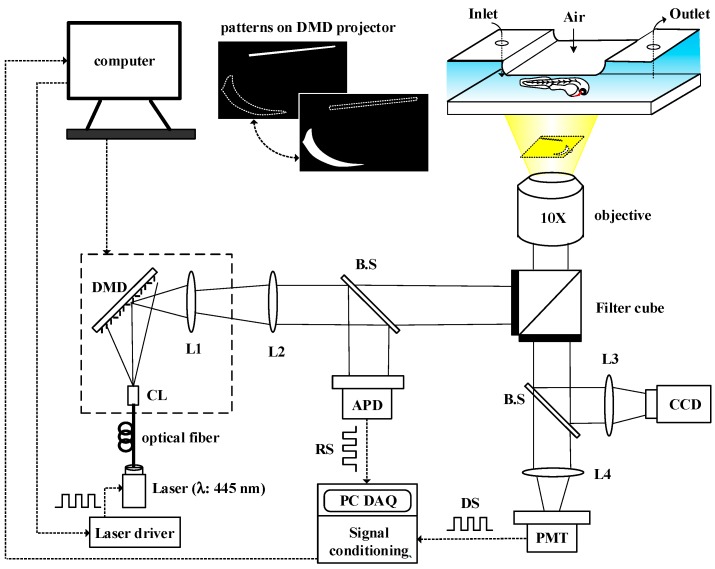
A schematic of the digital light modulation system utilizing a modified commercial DMD projector equipped with a fiber-guided laser diode as the light modulation source to spatially direct excited light toward a zebrafish embryo to detect *in vivo* tissue oxygenation in real time via phase-based phosphorescence lifetime detection. (CL: collimating lens; L1: built-in condensing lens; L2: projection lens; L3, L4: focus lenses; B.S: beam splitter; RS: reference signal; DS: detection signal; APD: amplified photodetector; and PMT: photomultiplier tube)

After immobilization, we can precisely project the illumination patterns toward to arbitrarily-shaped regions of tissue of interest for *in vivo* oxygen measurements without the need to realign between the optical patterns and the measured embryo. We introduced 0% or 20% pO_2_ embryo water into the microfluidic device and control the illumination pattern on the DMD to measure *in vivo* oxygen changes in real time in the “cardiac region (C.R.)” and “cardinal vein (C.V.)” of a 48 hpf zebrafish embryo that experience hypoxia (0% pO_2_) and subsequent normoxic conditions (20% pO_2_) with spatiotemporal resolution for distinct regional analysis. The *in vivo* oxygen change of C.R. and C.V. in a 48 hpf zebrafish embryo was performed by tracking the oxygen concentration over time via phase-based phosphorescence lifetime detection. To this end, we set up a digital-light modulation system utilizing a modified commercial DMD projector equipped with a fiber-guided laser diode as a light modulation source to modulate the excitation light in the spatial and temporal domains toward arbitrarily-shaped regions of tissue of interest in an unanesthetized zebrafish embryo to detect the oxygen concentration (Figure 2). The phosphorescence lifetime (τ) was calculated by measuring the phase shift (θ) between the reference (modulated excitation light) and the corresponding phosphorescent signals. The relationship between the oxygen concentration, phase shift (θ) and the lifetime (τ) is described in Section 2.3. 

### 2.2. DMD-Based Light Modulation System for Phase-Based Phosphorescence Lifetime Detection

Figure 2 shows the DMD-based light modulation system utilizing a modified commercial DMD projector (BENQ MP525P, Hsinchu, Taiwan) equipped with a fiber-guided laser diode (λ = 445 nm, Laser Star Inc., Taipei, Taiwan) for the light modulation source. To utilize the laser diode as a light modulation source, we modified the commercial DMD projector by simply removing the original lamp, the color filter wheel, and the original projection lens. The modulated laser diode was driven by an analog circuit to provide the excitation light at a rate of 5 kHz. The light patterns of the DMD were controlled by a computer. The modulated excitation light was directed into a standard inverted IX-71 fluorescence microscope (Olympus, Tokyo, Japan) toward the microfluidic device to illuminate arbitrarily-shaped regions of tissue of interest in an immobilized zebrafish embryo through lenses (L1 and L2), a filter unit (Ex: 420–480 nm; Em: 650 nm; Dm: 500 nm) and an objective lens with 4× or 10× magnification. The reference signal (RS) was recorded by measuring the light intensity of the modulated excitation light after passing through the DMD using an amplified photo-detector (ET-2030A, Electro-Optics Technology Inc., Traverse City, MI, USA) via a 50/50 beam splitter (B.S). The detection signal (DS) was recorded by simultaneously measuring the light intensity of the corresponding phosphorescence with a photo-multiplier tube (PMT-R928, Hamamatsu, Japan) and a cooled CCD camera (CoolSNAP HQ^2^, Photometrics, Tucson, AZ, USA) in real time along with the reference signal. The reference signal (RS) and the phosphorescent detection signal (DS) were both recorded on a computer via a USB DAQ card (USB-6251, National Instruments, Austin, TX, USA) and then followed by a signal conditioning process. The phosphorescence lifetime (τ) was calculated by measuring the phase shift (θ) between the reference signal (modulated excitation light) and the phosphorescent detection signal to calculate the luminescent lifetime (τ) via phase-based phosphorescence lifetime detection. The relationship between the phase shift (θ) and the lifetime (τ) can be approximated by the following:

(1) tan(θ)=2πντ

where ν is the modulation frequency. In this study, a modulation frequency of ν = 5 kHz was used to measure the luminescent lifetime for phase-based phosphorescence lifetime detection. The phase shift (θ) between the reference and detected signals was determined by digital lock-in analysis, as described in our previous work [27]. The phase shift (θ) was then used to calculate the luminescent lifetime (τ) according to Equation (1). The luminescent lifetime (τ) related to the oxygen pressure [pO_2_] is described by the Stern-Volmer relationship as follows:

(2) $$\frac{1}{\text{τ}}=\frac{1}{{\text{τ}}_{0}}+{\text{k}}_{\text{q}}[\text{p}{\text{O}}_{2}]$$

where τ_o_ is the lifetime measured in the absence of oxygen, ${\text{k}}_{\text{q}}$ is the quenching constant, and [pO_2_] is the oxygen pressure. 

### 2.3. Microinjection of Oxyphor G4 into a 48 hpf Zebrafish Embryo

Oxyphor G4 (Oxygen Enterprises Ltd., Philadelphia, PA, USA) is a type of phosphorescent probe (Pd-*meso*-tetra-(3,5-dicarboxyphenyl)-tetrabenzoporphyrin) that can operate in either albumin-rich (blood plasma) or albumin-free (interstitial space) environments at all physiological oxygen concentrations from normoxic to deep hypoxic conditions [8]. The initial concentration of the Oxyphor G4 solution is 200 µM after purchase. Oxyphor G4 is highly soluble in aqueous environments and do not extravasate from blood vessels. These properties make it very suitable for measuring tissue oxygenation which represents the oxygen concentrations in the microvasculature of tissue. 

**Figure 3:**
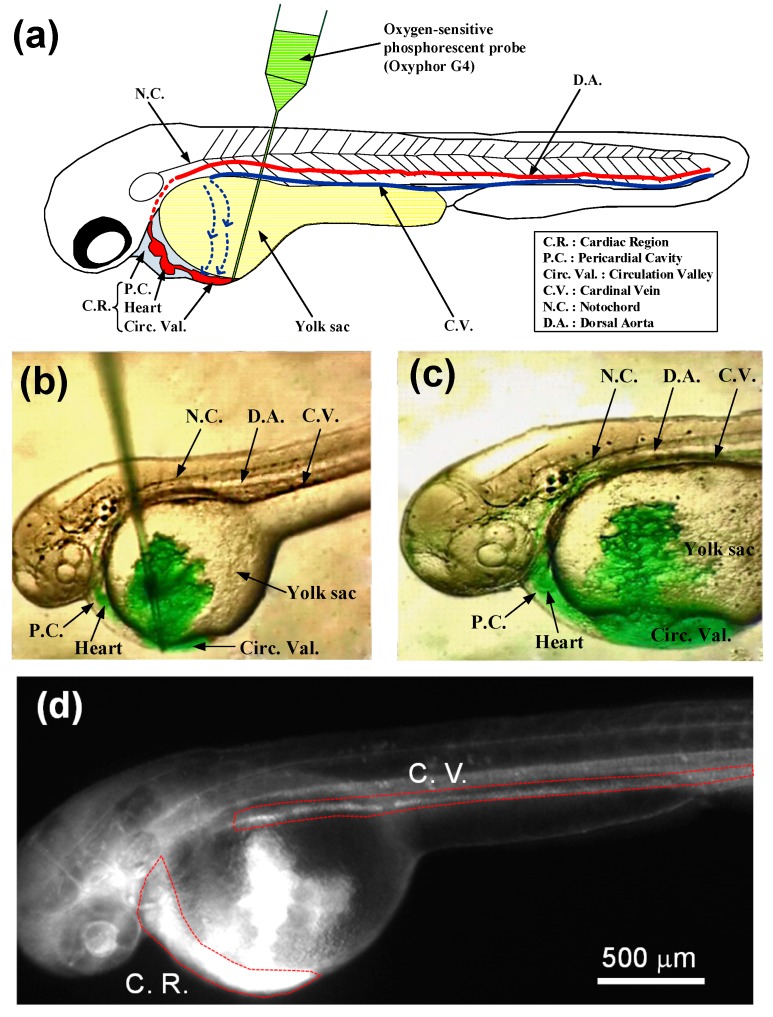
(**a**) A schematic of anatomical features of 48 hpf zebrafish embryos from lateral view, where the oxygen-sensitive phosphorescent probe (Oxyphor G4) was inoculated into the bloodstream via the circulation valley by using an glass microneedle held on micromanipulator and hydraulic microinjector; (**b**,**c**) Images of the 48 hpf zebrafish embryo during and after microinjection with a 9.2 nL of the Oxyphor G4 solution at 200 μM; (**d**) Phosphorescent image of the whole 48 hpf zebrafish embryo after microinjection.

This probe has been well calibrated in oxygen measurements under physiological conditions (pH 6.4–7.8) and temperatures (22–38 °C), showing high stability and reproducibility of phosphorescent signals [8]. Oxyphor G4 has an excitation peak near 448 nm and a peak emission near 813 nm. The measured lifetime (τ_0_) in the absence of oxygen is 242 μs and the value for kq is 190 mm Hg^−1^·s^−1^ at 22 °C and pH = 7.23. 

The fertilized zebrafish embryos were captured and maintained in an incubator at 28.5 °C according to standard methods in our laboratory [26]. We collected 48 hpf embryos for experimentation. Prior to microinjection, a 48 hpf zebrafish embryo was manually dechorionated and embedded in 0.8% low melt agarose (Sigma-Aldrich, St. Louis, MO, USA). The Oxyphor G4 was inoculated into the bloodstream of 48 hpf zebrafish embryos via the circulation valley located near to the yolk sac by using an injection glass microneedle held on micromanipulator and hydraulic microinjector (Nanoject II, Drummond Scientific Company, Broomall, PA, USA) as visually ascertained under a stereomicroscope as shown in Figure 3b,c. The circulation valley indicates an area where venous blood returning from the trunk and tail flows freely over the lateral sides of the yolk before returning to the heart. The dashed line with an arrowhead shown in Figure 3a indicates the direction of blood flow into the circulation valley. Inoculation into the circulation valley results in the rapid dispersion of the Oxyphor G4 into bloodstream throughout the embryo via blood circulation (see Supplementary Materials: Video). A volume of 4.6–18.4 nL of a dye stock solution (200 μM) was respectively microinjected into the circulation valley to choose the suitable injection volume, which can provide a sufficiently high phosphorescence signal without affecting the rhythmicity of the heart. Figure 3c shows the phosphorescent image of the whole 48 hpf zebrafish embryo after microinjection with a 9.2 nL of the Oxyphor G4 solution at 200 μM. Embryos after microinjection with Oxyphor G4 were examined every 3 h over the course of a 24 h period and scored for death, defined here as the complete absence of heart rhythm and blood flow.

### 2.4. Fabrication of Microfluidic Devices

The two-layered PDMS structures with microfluidic channels, which provided an entrance for fluid and high pressure air, were respectively fabricated using standard soft lithography. Poly(methyl methacrylate) (PMMA) molds with the negative pattern were first machined by a micro CNC machine. The PDMS pre-polymer and curing agent (Sylgard^®^ 184, Dow Corning, Midland, MI, USA) were mixed at 10:1 ratio. After stirred thoroughly and degassed in a vacuum chamber, the prepared PDMS mixture was used for the fabrication of both layers. The prepared PDMS mixture was spun at 500 rpm for 20 s poured on a plate to form 100 μm-thick membrane served as the pneumatically actuated PDMS membrane. After cured at 70 °C for 1 h, the PDMS microchannel for high pressure air was bonded onto the PDMS membrane using oxygen plasma treatment, and then was peeled off. Finally, the microfluidic device was assembled by bonding microchannels with thin membrane for high pressure air and microchannels for fluid together with proper alignment onto the glass substrate using oxygen plasma treatment (Figure 1b). The microchannels for fluid consists of an pumping port and reservoir output port with 3 mm diameter, and a straight channel 1 mm wide, 30 mm long and 0.5 mm tall, which connects the two ports with a constriction 0.3 mm wide towards the end (Figure 1c). The pneumatically actuated membrane with 10 mm long was set up covering the straight and constrictive microfluidic channels.

## 3. Results and Discussion

### 3.1. Survival Rates after Microinjection with Oxyphor G4

**Figure 4:**
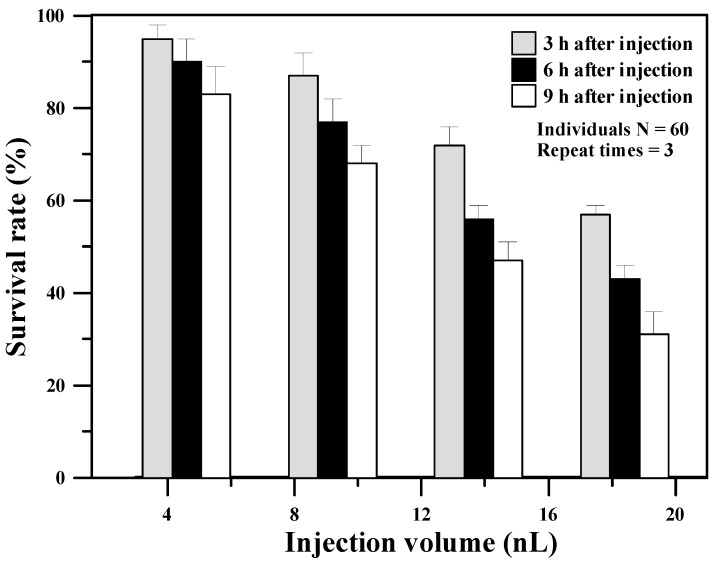
The survival rates (%) of 3–9 h after microinjecting 4.6, 9.2, 13.8, and 18.4 nL of 200 μM Oxyphor G4 solution into 48 hpf zebrafish embryos.

Although Oxyphor G4 has been previously used to measure *in vivo* oxygenation of murine tumors [8] or brain microvascular oxygenation in pigs during cardiac arrest [15] by inoculating Oxyphor G4 at a dose of 1–5 mg/kg body weight, one has never been used it to measure *in vivo* tissue oxygenation in zebrafish embryos. Therefore, we need to experimentally determine the suitable dose of Oxyphor G4 for zebrafish embryos to have higher survival rates after microinjection and also provide adequate intensity of phosphorescence for oxygen measurements. Figure 4 shows the survival rate (%) of 3–9 h after microinjecting 4.6, 9.2, 13.8, and 18.4 nL of 200 μM Oxyphor G4 solution into 48 hpf zebrafish embryos. The 3-h survival rates after microinjection at a dose less than 9.2 nL were found to be high (>80%), but significantly decreased with increasing the injection volumes, such as only 57% survival for microinjection at 18.4 nL. The 9-h survival rate after microinjection at a dose of 9.2 nL was about 68%, but only 47% and 31% survival for microinjection at 13.8 nL and 18.4 nL, respectively. Although the 9-h survival rate (78%) at a dose of 4.6 nL was higher than that (68%) at 9.2 nL, the lower phosphorescent intensity due to the less injection dose (4.6 nL) significantly decreased the S/N ratio during oxygen measurements. Therefore, the suitable dose of 9.2 nL Oxyphor G4 solution at 200 μM was chosen in this study to maintain acceptable survival rates after microinjection and provide adequate intensity of phosphorescence for measurements of *in vivo* tissue oxygenation in a zebrafish embryo. 

### 3.2. Physiological States of Zebrafish Embryos after Immobilization

**Figure 5:**
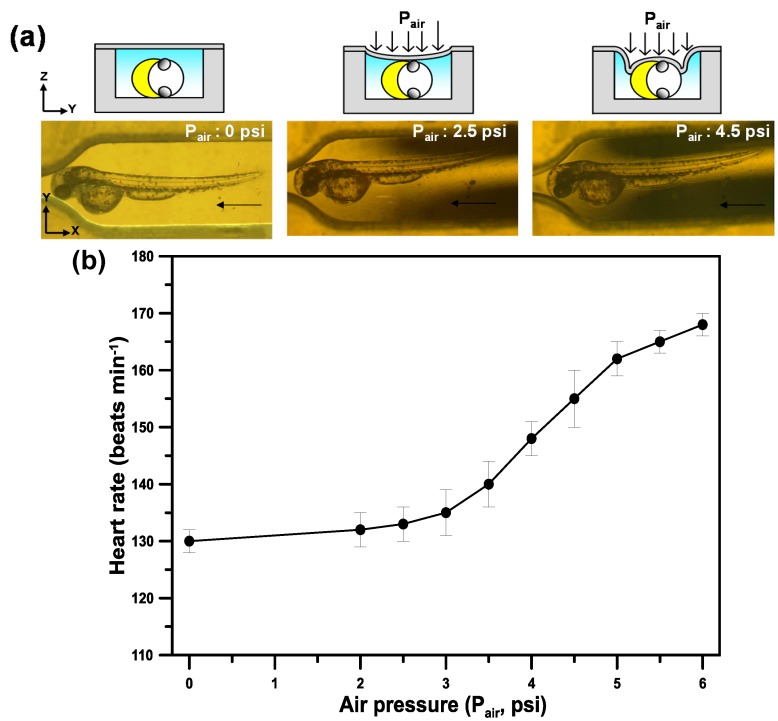
(**a**) The optical images and (**b**) the heart rate (beats min^−1^) of 48 hpf zebrafish embryos immobilized on its lateral side by using a pneumatically actuated membrane at different air pressures.

We introduced the unanesthetized zebrafish into the microfluidic device, positioned and immobilized on its lateral side by using a pneumatically actuated membrane. Although the immobilization of the unanesthetized zebrafish embryo is necessary in our experiments to prevent from free movement of the unanesthetized zebrafish embryo during *in vivo* oxygen measurements, we need to verify that the immobilization did not adversely affect the physiological states of the measured 48 hpf zebrafish embryos. Figure 5 shows the optical images and the heart rate (beats min^−1^) of 48 hpf zebrafish embryos immobilized on its lateral side by using a pneumatically actuated membrane at different air pressures. After introducing an unanesthetized zebrafish embryo head-first by using passive pumping, we observed an unanesthetized zebrafish embryo trapped by constrictive microfluidic channels (P_air_ = 0 psi in Figure 5a left). The heart rate of the unanesthetized zebrafish embryo without immobilization by a pneumatically actuated PDMS membrane was 130 beats min^−1^ at a normal physiological state. Heart rate was determined by measuring the time interval for 30 heart beats with triplicate measurements. An air pressure (P_air_) was used to pneumatically actuate the PDMS membrane to immobilize the unanesthetized embryo on its lateral side. At P_air_ = 2.5 psi (17.2 kPa), the unanesthetized zebrafish embryo was slightly pressed on its partly lateral side by the PDMS membrane as shown in Figure 5a middle. 

The heart rate of the zebrafish embryo immobilized at P_air_ = 0 psi was about 133 beats min^−1^ indicating that the slightly pressed force on the zebrafish embryo did not adversely affect the physiological state of the measured embryo. At P_air_ = 4.5 psi (31 kPa), the zebrafish embryo was greatly pressed on its whole lateral side by the PDMS membrane as shown in Figure 5a right. The heart rate of the zebrafish embryo increased to 155 beats min^−1^ (Figure 5b) indicating that the physiological state of the measured embryo was significantly affected at this high pressed force. As shown in Figure 5b, increasing the pressed pressure (P_air_) resulted in an increase of the heart rate of 48 hpf zebrafish embryos immobilized by using a pneumatically actuated membrane. The pressed pressure at P_air_ = 3 psi (20.7 kPa) was chosen in this study to maintain the unanesthetized 48 hpf zebrafish embryo at its normal physiological state after immobilization and provide adequate space without blockage by the pneumatically actuated membrane, which enables us to perfuse normoxic/hypoxic embryo medium into a microfluidic device for oxygen measurements under HR.

### 3.3. Phase-Based Phosphorescence Lifetime Detection for Oxygen Measurements

**Figure 6:**
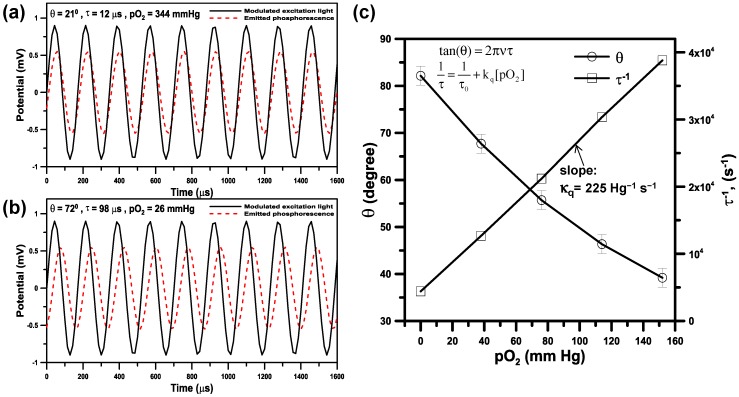
The typical time domain of the reference signal (modulated excitation light) and the corresponding phosphorescent detection signal for oxygen measurements at (**a**) pO_2_ = 344 mmHg and (**b**) pO_2_ = 26 mmHg, respectively; (**c**) Calibration on the phase shift (θ), a reciprocal of the luminescent lifetime (τ) with respective to the oxygen pressure [pO_2_].

Figure 6a,b show the typical time domain of the reference signal (modulated excitation light) and the corresponding phosphorescent detection signal for oxygen measurements at pO_2_ = 344 mm Hg (45% pO_2_) and 26 mm Hg (3% pO_2_), respectively. A phase shift (θ) was clearly observed between the reference signal and the corresponding detection signal. For the 5 kHz excitation and emission signals, the phase shift (θ) was determined by the digital lock-in detection in the Labview module and then used to calculate the luminescent lifetime (τ) according to Equation (1). 

The oxygen pressure [pO_2_] related to the luminescent lifetime (τ) was calculated by the Stern-Volmer relationship (Equation (2)). Calibration tests on the phase shift (θ), a reciprocal of the luminescent lifetime (τ) with respective to [pO_2_] were performed by introducing solution of the probe (Oxyphore G4) to a sealed reservoir and then bubbling with N_2_ gas for a minimum of 10 min to respectively provide [pO_2_] ranging from 0 to 152 mm Hg (Figure 5c). The oxygen level in the reservoir was continuously monitored by Clarke microelectrode sensors (DO-5510, LUTRON, Taipei, Taiwan). In our Stern-Volmer calibration curve in Figure 5c, a nearly linear relationship was observed between the reciprocal of lifetime (τ) and the [pO_2_]. The measured lifetime (τ_o_) via phase-based (frequency-domain) phosphorescence lifetime detection in the absence of oxygen is 231 μs and the value for k_q_ is 225 mm Hg^−1^·s^−1^ at 28 °C and pH = 7.6, which are close to the values for τ_o_ = 242 μs and k_q_ = 190 mm Hg^−1^·s^−1^ measured by the time-domain phosphorescence lifetime detection for Oxyphore G4 at 22 °C and pH = 7.23 in the previous work [8].

### 3.4. Light-Addressable Measurement of In Vivo Tissue Oxygenation

To test the ability of our proposed light-addressing projection system to illuminate arbitrarily-shaped regions of tissue of interest for *in vivo* oxygen measurements, we controlled the illumination pattern on the DMD to spatially project the modulated excitation light on the cardiac region (C.R.) and cardinal vein (C.V.) of a 48 hpf zebrafish embryo for *in vivo* oxygen measurements. The C.R. includes the pericardial cavity (gray color in Figure 3a), heart and circulation valley (red color in Figure 3a). The C.R. and C.V. of a 48 hpf zebrafish were chosen for *in vivo* oxygen measurements in this study due to its fast response to environmental oxygen changes. 

**Figure 7:**
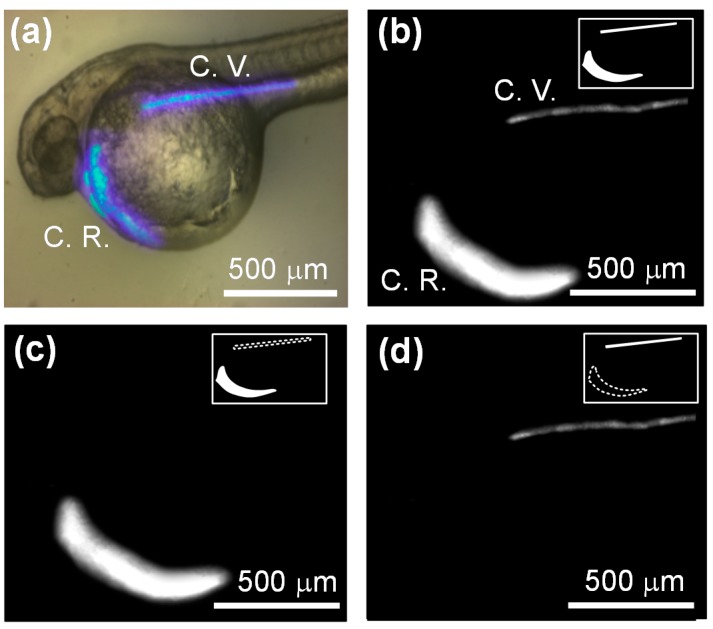
(**a**) The optical image of a 48 hpf zebrafish at a normoxic condition (20% pO_2_) when the illumination pattern was spatially projected to simultaneously excite the C.R. and C.V. regions; The phosphorescent images of the C.R. and C.V. regions of a 48 hpf zebrafish that was excited by dynamically addressing modulated light patterns to simultaneously excite (**b**) both regions or (**c**,**d**) only excite the individual region for *in vivo* oxygen measurements. The inserts on the upper-right of the images indicate the pattern on the DMD.

Figure 7a shows an optical image of a 48 hpf zebrafish at a normoxic condition (20% pO_2_) when the illumination pattern was spatially projected to simultaneously excite the C.R. and C.V. regions. Figure 7b,c show phosphorescent images of the C.R. and C.V. regions of a 48 hpf zebrafish that was excited by dynamically addressing modulated light patterns to simultaneously excite both regions or only excite the individual region for *in vivo* oxygen measurements via phase-based phosphorescence lifetime detection. The whole projected area on the glass substrate was about 6 mm × 5 mm with a spatial resolution of 1024 × 768 pixels through 10× objective lens. To achieve acceptable phosphorescent signals for oxygen measurements, the minimum excitation area was about 0.1 mm × 0.1 mm. In our developed DMD-based light modulation system, the temporal resolution of the [pO_2_] was 0.5 s at each illumination region, where the [pO_2_] was calculated by averaging 10 successive phase shift (θ) values. We can dynamically scan two different regions per second and collect the phosphorescence signal in each illumination region for *in vivo* oxygen measurements by controlling the light patterns of the DMD. The [pO_2_] of the C.R. and C.V. were about 96 mm Hg and 77 mm Hg at a normoxic condition, respectively. We also measured the individual regions of heart, P.C. and Circ. Val. within the C.R. by changing the corresponding light patterns, respectively. The discrepancy in [pO_2_] between these individual regions was less than 5%, which was close to the [pO_2_] of the C.R. These results demonstrated the ability of the proposed DMD-based light modulation system to independently measure the local [pO_2_] by sequentially addressing an individual region of interest with spatiotemporal resolution for distinct regional analysis.

### 3.5. In vivo Tissue Oxygenation under Hypoxia/Reperfusion (HP)

**Figure 8:**
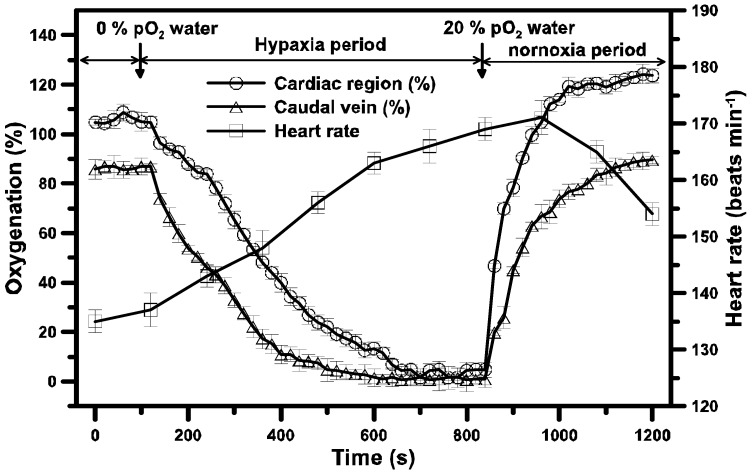
The time variation of the heart rate (beats min^−1^) and *in vivo* oxygen changes (%) in C.R. and C.V. of a 48 hpf zebrafish embryo that experience hypoxia (0% pO_2_) and subsequent normoxic conditions (20% pO_2_) by sequentially perfusing normoxic/hypoxic/normoxic medium into a microfluidic channel.

Figure 8 shows the time variation of the heart rate (beats min^−1^) and *in vivo* oxygen changes (%) in C.R. and C.V. of a 48 hpf zebrafish embryo that experience hypoxia (0% pO_2_) and subsequent normoxic conditions (20% pO_2_) by sequentially perfusing normoxic/hypoxic/normoxic medium into a microfluidic channel. At a normoxic condition (150 mm Hg, 20% pO_2_), the [pO_2_] of the C.R. and C.V. maintained at a stable value of 96 mm Hg and 77 mm Hg, respectively.

Obviously, after supplying oxygen to tissues and organs along the central circulatory system, the oxygen of the venous blood in C.V. reduced to about 80% compared to that in C.R. The heart rate of the 48 hpf zebrafish embryo was 137 beats min^−1^ at a normal physiological state. After perfusing hypoxic medium (0% pO_2_) into a microfluidic channel, we observed the progressive reduction in oxygen for C.R. and C.V. The [pO_2_] of C.R. and C.V. gradually dropped to 0 mm Hg (~100% reduction compared to normoxic condition) after 10 min and 8 min under acute hypoxia. However, the heart rate gradually increased to over 170 beats min^−1^. The increase of the heart rate results from an increase of cardiac activity in order to stimulate the convective oxygen transport for a suitable response under acute hypoxia. The stimulation of cardiac activity and significant drop of *in vivo* oxygen within few minutes induced by acute hypoxia for a 48 hpf zebrafish embryo in our experiments are consistent with the previous work verified by hemoglobin oxygen saturation using a spectrophotometrical analysis [28]. After the C.R. and C.V persisted for 11 min at 0% pO_2_ under hypoxia, we perfused normoxic medium (20% pO_2_) into a microfluidic channel. The progressive increase in oxygen for C.R. and C.V to the initial condition (100%) was observed after 3 min under normoxia. It seems that it took more time for the 48 hpf zebrafish embryo to respond to the transition from normoxia to hypoxia than that from hypoxia to normoxia. The heart rate gradually reduced from 172 to 153 beats min^−1^ after the transition from hypoxia to normoxia after 5 min, which was still larger than 137 beats min^−1^ at an initially normal physiological state. The large heart rate stimulated the oxygen transport to increase *in vivo* oxygen of the C.R. and C.V up to 120% and 90% at 1200 s after recovery at normoxic condition. More time was needed to entirely recover to a normal physiological state from hypoxia to normoxa.

## 4. Conclusions

In this study, we developed a digital light modulation system that utilizes a modified commercial DMD projector equipped with a laser diode as a light source to modulate the excitation light in the spatial and temporal domains for quantitative measurements of *in vivo* tissue oxygenation in an unanesthetized zebrafish embryo in real time via phase-based phosphorescence lifetime detection. The unanesthetized zebrafish embryo was introduced into the microfluidic device and immobilized on its lateral side by using a pneumatically actuated membrane. The anesthetization of the measured zebrafish embryo is no longer needed to prevent this from interfering with its physiological state. A suitable dose of 9.2 nL Oxyphor G4 solution at 200 μM was experimentally determined to maintain zebrafish survival after microinjection and provide adequate intensity of phosphorescence for oxygen measurements. We successfully measured *in vivo* oxygen changes in the C.R. and C.V. of a 48 hpf zebrafish that experience hypoxia and subsequent normoxic conditions with spatiotemporal resolution for distinct regional analysis. To the best of our knowledge, this is the first work to demonstrate *in vivo* oxygen measurements for physiologically relevant whole organism model, the zebrafish embryo, by phosphorescence quenching via phase-based phosphorescence lifetime detection. Currently, we are ongoing to measure *in vivo* oxygen changes in zebrafish eye and brain tissues to investigate the correlation between oxygen distribution and apoptotic cell death in these tissue microvasculature under HP injury by using terminal deoxynucleotidyl transferase dUTP nick end labeling (TUNEL) analysis. Our proposed platform provides the potential for the real-time investigation of oxygen distribution in tissue microvasculature that relates to physiological stimulation and diseases in a developing organism.

## Supplementary Files

Supplementary File 1
